# Preoperative BChE serves as a prognostic marker in patients with resectable AEG after neoadjuvant chemotherapy

**DOI:** 10.1007/s00423-023-02938-w

**Published:** 2023-06-06

**Authors:** Lisa Gensthaler, Gerd Jomrich, Jonas Brugger, Dagmar Kollmann, Matthias Paireder, Milena Bologheanu, Alexander Horn, Franz M. Riegler, Reza Asari, Sebastian F. Schoppmann

**Affiliations:** 1 Division of Visceral Surgery, Department of General Surgery, Comprehensive Cancer Center Vienna, Upper GI-Service, Medical University of Vienna, Spitalgasse 23, 1090 Vienna, Austria; 2 Section for Medical Statistics (IMS), Center of Medical Statistics, Informatics and Intelligent Systems, Medical University of Vienna, Spitalgasse 23, 1090 Vienna, Austria; 3 Reflux Ordination, Mariannengasse 10/4/9, 1090 Vienna, Austria; 4 Department of General Surgery, Medical University of Vienna, Waehringer Guertel 18-20, 1090 Vienna, Austria

**Keywords:** Adenocarcinoma of the gastroesophageal junction (AEG), Prognosis, Oncologic treatment, Biomarker, Butyrylcholinesterase (BChE)

## Abstract

**Background:**

Diminished systemic serum butyrylcholinesterase (BChE), a biomarker for chronic inflammation, cachexia, and advanced tumor stage, has shown to play a prognostic role in various malignancies. The aim of this study was to investigate the prognostic value of pretherapeutic BChE levels in patients with resectable adenocarcinoma of the gastroesophageal junction (AEG), treated with or without neoadjuvant therapy.

**Methods:**

Data of a consecutive series of patients with resectable AEG at the Department for General Surgery, Medical University of Vienna, were analyzed. Preoperative serum BChE levels were correlated to clinic-pathological parameters as well as treatment response. The prognostic impact of serum BChE levels on disease-free (DFS) and overall survival (OS) was evaluated by univariate and multivariate cox regression analysis, and Kaplan–Meier curves used for illustration.

**Results:**

A total of 319 patients were included in this study, with an overall mean (standard deviation, SD) pretreatment serum BChE level of 6.22 (± 1.91) IU/L. In univariate models, diminished preoperative serum BChE levels were significantly associated with shorter overall (OS, *p* < 0.003) and disease-free survival (DFS, *p* < 0.001) in patients who received neoadjuvant treatment and/or primary resection. In multivariated analysis, decreased BChE was significantly associated with shorter DFS (HR: 0.92, 95% CI: 0.84–1.00, *p* 0.049) and OS (HR: 0.92, 95% CI: 0.85–1.00, *p* < 0.49) in patients receiving neoadjuvant therapy. Backward regression identified the interaction between preoperative BChE and neoadjuvant chemotherapy as a predictive factor for DFS and OS.

**Conclusion:**

Diminished serum BChE serves as a strong, independent, and cost-effective prognostic biomarker for worse outcome in patients with resectable AEG who had received neoadjuvant chemotherapy.

## Introduction

Esophageal cancer is the sixth most common cause of cancer-related death worldwide with poor 5-year overall survival of approximately 30% [1, 2]. Especially the incidence of adenocarcinomas of the esophagogastric junction (AEG) has increased steadily over the past two decades. Since lifestyle-mediated predisposing risk factors, such as gastroesophageal reflux disease (GERD) and obesity, are a major trigger for the development of AEG, well-developed countries such as Northern America and Europe have been affected especially [3, 4]. In Western countries, AEG accounts for almost 60% of all esophageal cancers [5, 6], affecting mainly men (4.87%/100.000) between the age of 45 and 65 [7]. Due to enhanced morbidity and mortality, early detection and precise treatment in specialized, high-volume centers by specialized surgeons are known to improve patients’ outcome significantly [8]. Therefore, preventive and early screening methods to detect patients at risk have to be implemented. Several clinicopathological parameters, such as age at diagnosis, tumor stage and grading, histological subtype, and lymph node involvement as well as the presence or absence of ascites, have been proposed as predictors for overall survival in patients with esophageal cancer [9–12]. Since few prognostic factors are available prior surgery that indicate prognosis and treatment response, implementation of further practicable and convenient prognostic tools is highly required. Chronic inflammation is leading to metabolic alterations, muscle wasting, and cachexia and is known as predictor in tumor initiation [13]. Furthermore, several serological biomarkers are potentially reflecting a state of inflammation and malnutrition and have been linked to the oncologic outcome of patients with esophageal cancer [14–17]. While the association of malnutrition and poor oncologic outcome can be evaluated by using the controlling nutritional status (CONUT) score [18], chronic inflammation and its impact on the prognosis in esophageal cancer patients were discussed by evaluating the preoperative neutrophil-to-lymphocyte ratio (NLR) or the albumin-to-fibrinogen ratio, for instance [19, 20]. Butyrylcholinesterase (BChE) is a non-specific enzyme, synthetized by the liver and found in most tissues, including the nervous system, small intestine, and adipocytes [21]. Decreased serum BChE was observed in patients with chronic inflammation, malnutrition, and malignant disease, serving for instance as a potential biomarker for organ damage and impaired liver function [22]. Furthermore, an association of decreased BChE levels and advanced tumor stage, cancer cachexia, and poor survival in patients suffering from other gastrointestinal, gynecologic, and urologic malignancies has been described earlier [21, 23–25]. However, the role of BChE as a prognostic parameter in patients with AEG has not been evaluated so far. The aim of this study was to investigate the prognostic value of preoperatively obtained serological BChE on patients’ DFS and OS with resectable AEG with or without neoadjuvant chemotherapy in a large consecutive single center cohort.

## Materials and methods

Between 1992 and 2016, 544 patients were diagnosed with resectable AEG at the Medical University of Vienna, Department for Surgery. Patients’ records were examined to identify and include those with available serological measurements of BChE before treatment (neoadjuvant chemotherapy and concomitant surgery or surgery alone). Clinicopathological data was collected from the local prospective database and analyzed retrospectively. Therefore, data of 319 patients could be included in this retrospective, single center cohort study. Tumor location was diagnosed endoscopically or radiologically with computed tomography scan (CT scan) and categorized using the classification of Siewert and Stein [26]. Using the tumor-node-metastasis classification of the Union for International Cancer Control (UICC), clinical and pathologic classification of the primary tumor, lymph node involvement, and presence of distant metastasis were defined (cTNM, pTNM). As part of the clinical routine checkup, patient’s blood was obtained between 24 and 72 h prior surgery or neoadjuvant therapy, respectively, by peripheral venous puncture and quantitative values of serum BChE were assessed. Serum samples were analyzed at the laboratory of the Medical University of Vienna, using VITROS® MicroSlide Technology; quality was guaranteed by frequent controls of analytical and functional methods and calibration. Patients’ comorbidities, such as impaired liver function, were assessed via anamnesis, blood analysis, and examination of a specialist in internal medicine and an anesthetist prior to surgery. Only patients in acceptable clinical and physical condition were considered eligible for surgery. Abdominothoracic esophageal resection was performed in patients with AEG 1 or 2, and transhiatal extended gastrectomy was the surgical method of choice in case of AEG 2 or 3. Patients with resectable AEG, cT1, N1-2, or cT2-4a, N0-3 received neoadjuvant treatment based on defined treatment regimen by the institution’s interdisciplinary tumor board. Regimen A, a combination of oxaliplatin and capecitabine, or regimen B, cisplatin and 5-fluoruracil, was administered intravenously as neoadjuvant chemotherapy. According to the recommendations of the interdisciplinary tumor board, concomitant radiation (regimen C) was performed based on the publication by Van Hagen et al. in some cases [27]. Mandard response was applied as a tool to define response to neoadjuvant treatment [28]. Additionally, effects of age, BMI (< 25 vs. ≥ 25 kg/m^2^), tumor localization (AEG 1 vs. 2 vs. 3), response to treatment (Mandard response 1 vs. 2 vs. 3 vs. 4), tumor size (cT1 vs. 2 vs. 3 vs. 4), lymph node involvement represented by the lymph node ratio (< 0.3 vs. ≥ 0.3), and histological grading (G1 vs. G2 vs. G3) on survival probabilities were assessed. Within the first 2 years after surgery, patients were observed regularly every 3 months via physical examination, CT scan, and follow-up of tumor marker blood samples, followed by observation every 6 months within the 2nd–5th year after surgery. Exclusion criteria were missing preoperative serum BChE levels and missing follow-up data, patients receiving chemotherapy exclusively, and distant metastasis at the time of surgery; patients with coexisting malignancies other than AEG, insufficient resection margin, and postoperative death by any cause other than AEG within 30 days after surgery were excluded from analysis as well. Patient’s data were retrospectively collected, pseudonymized, and handled carefully regarding standards of Good Scientific Practice. Since no additional examinations or tests during the perioperative period were required, no informed consent was necessary. Before initiation, the study was approved by the institutional ethics committee of the Medical University of Vienna, Austria, according to the declaration of Helsinki (IRB number: 2251/2019).

### Statistical evaluation

Categorical variables are presented as numbers (*n*). Continuous variables are presented as mean (SD). With regard to the type of treatment (neoadjuvant therapy vs. surgery alone), we performed univariable Cox regression with disease-free survival and overall survival as endpoints and the potential predictive factors, respectively, as explanatory variables. Survivors were censored at the last date they were known to be alive. *p*-values of continuous or dichotomous parameters were computed using Wald statistics. Statistical significance of discrete parameters with more than two levels was evaluated using likelihood-ratio tests. We computed multivariable cox regression models with disease-free survival and overall survival as endpoints and defined as explanatory variables the parameters that were statistically significant in the univariable models and an interaction term between treatment and preoperative BChE. We computed *p*-values of the respective variables analogous to the univariable procedure and performed post hoc comparisons for discrete factors. As a sensitivity analysis, we performed backwards regression using the Akaike information criterion (AIC) as the information criterion on both the multivariable models to identify the best predictive factors. pN was excluded from all multivariable analyses due to its high number of missing values. Additionally, we performed Kaplan–Meier curves with patients grouped based on the empirical quartiles of BChE to illustrate its effect on the observed survival times. The significance level was defined to be *α* = 0.05. However, no correction for multiple testing was applied; therefore, all *p*-values are of descriptive character. Statistical analysis was done using SPSS 25.0 (SPSS 25.0, IBM Inc., Armonk, NY) and R, version 4.2.0 or higher.

## Results

**Fig. 1:**
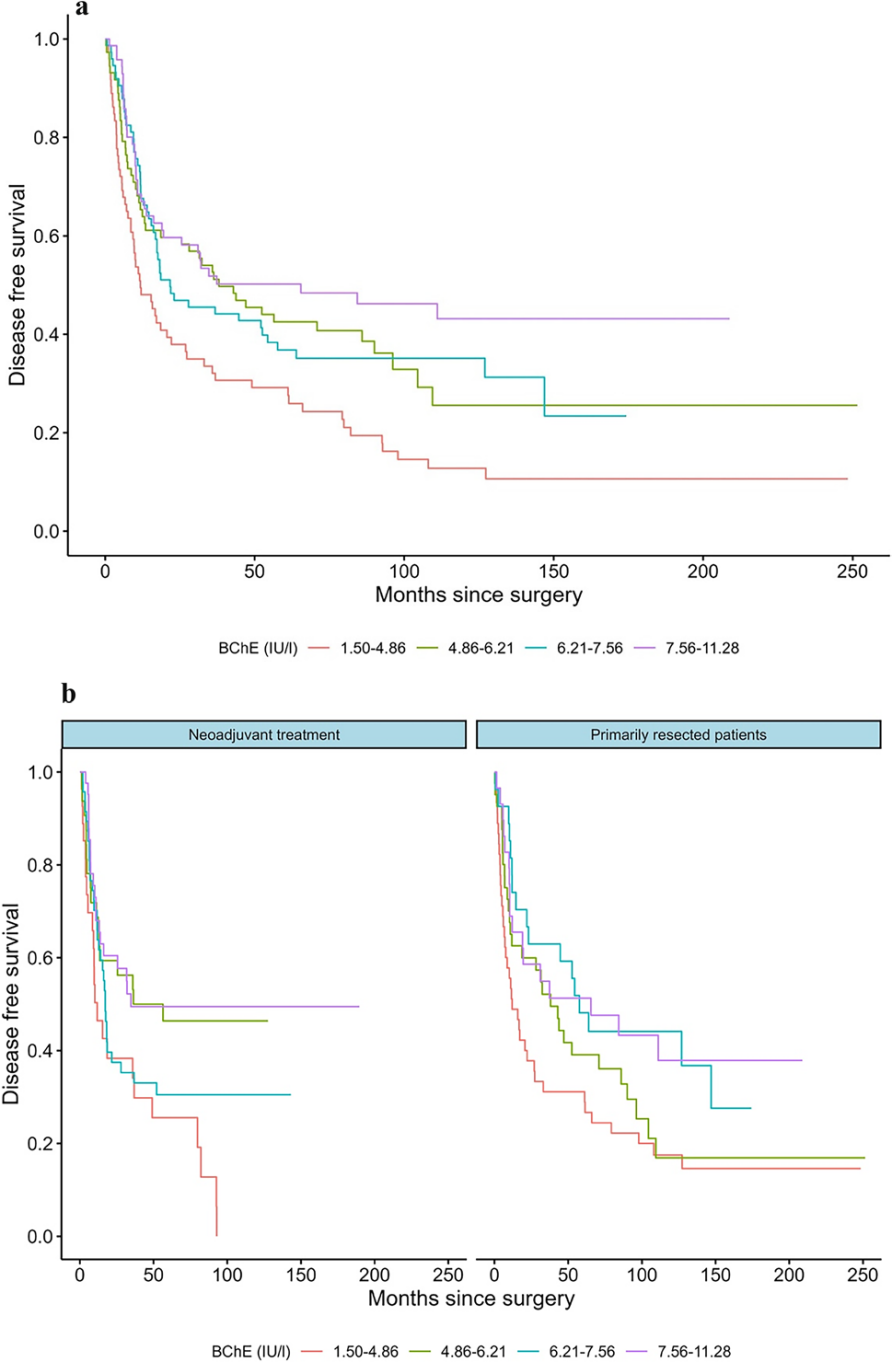
**a**, **b** Kaplan–Meier curves for disease-free survival in all patients with AEG stratified by BChE quartiles (**a**). **b** Disease-free survival in patients receiving neoadjuvant therapy (yes/no) and primarily resected patients with regard to BChE, *n* = 319

**Fig. 2:**
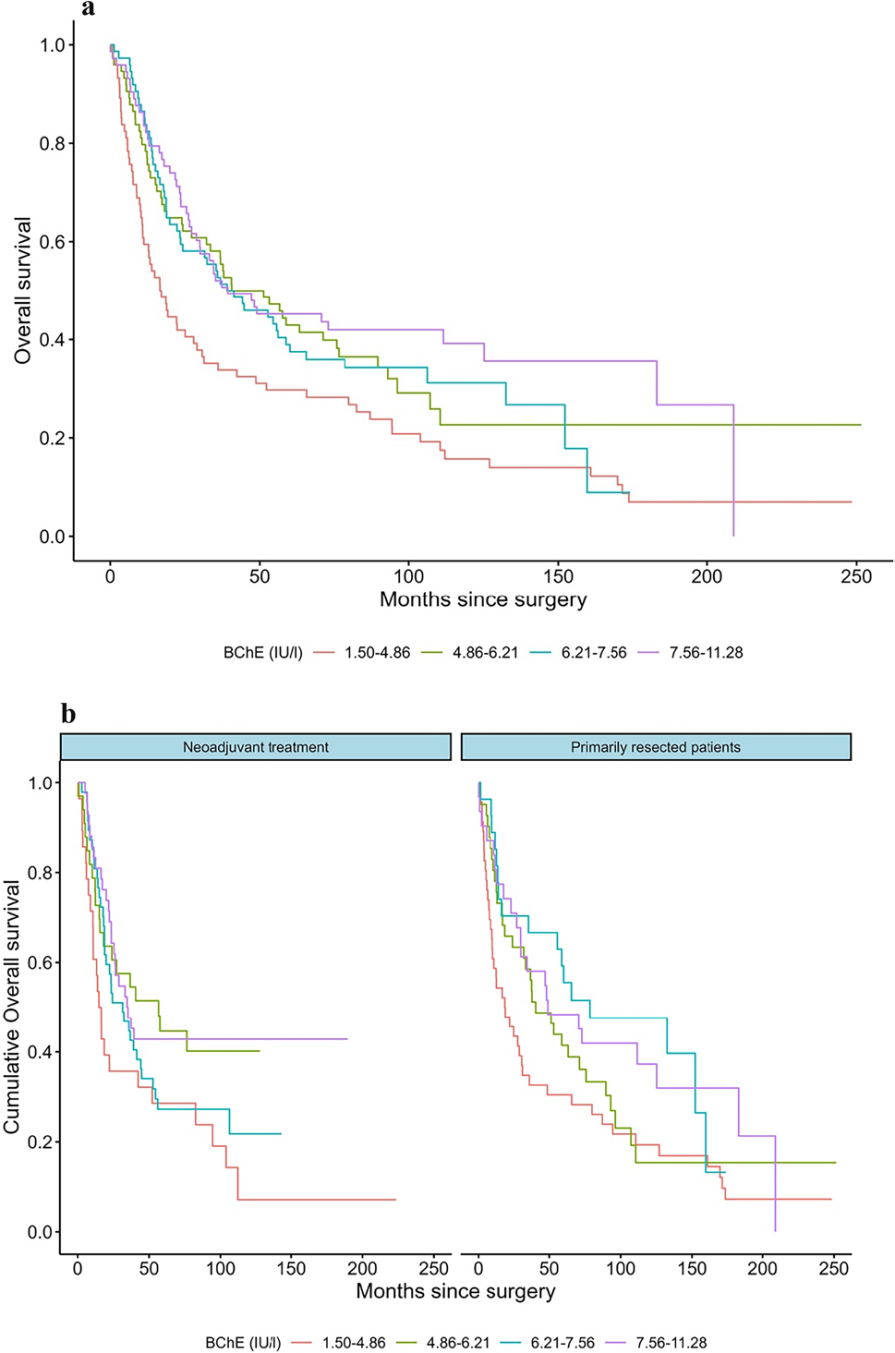
**a**,** b** Kaplan–Meier curves for overall survival in all patients with AEG stratified by BChE quartiles (**a**). **b** Overall survival in patients receiving neoadjuvant therapy (yes/no) and primarily resected patients with regard to BChE, *n* = 319

A total of 544 patients, diagnosed with resectable AEG between 1992 and 2016 at the Department for Surgery, Medical University of Vienna, were identified and included in our prospective database. Data on preoperative serological parameters of BChE were available in 319 patients and, therefore, considered eligible for further investigation. Cohorts of patients receiving neoadjuvant treatment (group A, *n* = 158; 49.5%) and those undergoing primary resection (group B, *n* = 161; 50.5%) were distributed similarly. Mean age at the time of surgery was 63.9 (± 10.8) years. The study included 259 (81.2%) male and 60 (18.8%) female patients. In patients with neoadjuvant treatment, the mean body mass index (BMI) was 29.2 kg/m^2^ prior to surgery, and in those without neoadjuvant treatment 26.5 kg/m^2^. BMI was significantly associated with DFS in univariate analysis. Of the 158 (49.4%) patients who received neoadjuvant treatment, the tumor was diagnosed as AEG I in most cases (*n* = 108, 68.4%), whereas tumor localization was more evenly distributed in the primarily resected group (AEG I: *n* = 76 (47.2%), AEG II: *n* = 77 (47.8%)). Ninety-three patients (58.9%) received a combination of oxaliplatin and capecitabine (regimen A) as neoadjuvant therapy, 55 (34.8%) cisplatin and 5-fluoruracil (regimen B), and 10 (6.3%) concomitant radiation (regimen C). 4.1% (*n* = 13) of patients showed a complete response (CR) after neoadjuvant treatment. Prior to treatment, the mean serum BChE level of all patients was 6.22 (± 1.91) IU/L. In patients receiving neoadjuvant chemotherapy, pretherapeutic mean serum BChE level was 7.18 (± 3.27) and dropped to 6.51 (± 1.75) preoperatively within the observational period (Δ − 0.6). Mean values of routinely assessed liver enzymes such as GGT (gamma-glutamyl-transferase) and LDH (lactate dehydrogenase) prior to systemic therapy and surgery were evaluated as well and were within the range of reference levels. Abdominothoracal surgery was the preferred surgical approach in both groups. For all patients, median overall survival (OS) was 35.4 months, and median time to recurrence (DFS) 20.6 months. OS and DFS in patients receiving neoadjuvant therapy were shorter than in the primarily resected group (Table 1). In the univariable models, preoperative BChE (*p* < 0.001), clinical tumor stage (*p* < 0.001), preoperative UICC classification (*p* < 0.001), grading (*p* < 0.001), pT (*p* < 0.001), pN (*p* < 0.017), and lymph node ratio (*p* < 0.001) were significantly associated with disease-free survival. Regarding analyses with overall survival as endpoint, BChE (*p* < 0.003), clinical tumor stage (*p* < 0.001), preoperative UICC classification (*p* < 0.001), grading (*p* < 0.001), pT (*p* < 0.001), pN (*p* < 0.003), and lymph node ratio (*p* < 0.001) were significantly related. Demographic and descriptive data of the patient population and clinicopathologic characteristics as well as univariable analysis regarding disease-free and overall survival as endpoint are given in Table 1. Multivariate Cox regression analysis on the influence of BChE and clinicopathologic parameters on DFS and OS for patients receiving neoadjuvant therapy and surgery alone was performed using the predictive factors that were significant in the univariable models and the interaction of treatment and preoperative BChE (*n* = 319; Table 2). Interestingly, in multivariable analysis, preoperative BChE was significantly associated with shorter disease-free survival in patients with neoadjuvant chemotherapy (*p* < 0.049, HR: 0.92, 95%CI: 0.84–1.00), also regarding OS (*p* < 0.049, HR: 0.92, 95% CI: 0.85–1.00). In the subgroup of primarily resected patients, BChE levels were also estimated to be associated with shorter survival times. However, the relationship was not statistically significant (DFS: *p* < 0.35, HR: 0.96, 95%CI: 0.87–1.05; OS: *p* < 0.045, HR: 0.97, 95%CI: 0.89–1.06). The lymph node ratio (LNR) remained statistically a highly significant parameter regarding DFS (*p* < 0.001, HR: 4.99, 95% CI: 2.52–9.90) and OS (*p* < 0.001, HR: 5.42, 95% CI: 2.81–10.44) in multivariate analysis (Table 2). Interestingly, besides pathologic tumor stage (pT) in OS, no other parameters remained statistically significant in multivariate analysis. In a sensitivity analysis, backwards regression identified the interaction between preoperative BChE and neoadjuvant chemotherapy (*p* = 0.04, HR 0.92, 95% CI: 0.84–1.0), tumor stage, and lymph node ratio as the most important predictive factors for DFS. Similarly, the backwards regression recognized the same factors as the principal predictive markers for OS (*p* = 0.05, HR 0.92, 95% CI: 0.85–1.0) (Table 3). Empirical differences in survival time with regard to preoperative BChE and treatment are illustrated in Figs. 1a and b and 2a and b. Patient data was grouped based on the empirical quartiles of BChE levels.

**Table 1:** Descriptive data of the final patient population. Clinicopathologic characteristics and *p*-values of univariable cox regression models with disease-free and overall survival as endpoints

Parameters	Neoadjuvant chemotherapy Group A		Primary surgery Group B		DS	OS
	*n*	%	*n*	%	*p*	*p*
	158	49.5	161	50.5		
Age (years, mean ± SD)	62.0 (± 10.6)		65.8 (± 10.7)		0.7	0.5
Sex					-	-
Male	133	84.2	126	78.3		
Female	25	15.8	35	21.7		
BMI preoperative (mean)	29.2 kg/m^2^		26.5 kg/m^2^		0.05	0.17
BChE (mean ± SD)					< 0.001	0.003
Prior neoadjuvant therapy	7.18 (± 3.3)		-			
Prior Surgery	6.51 (± 1.75)		5.92 (± 2.03)			
AEG					-	-
I	108	68.4	76	47.2		
II	31	19.6	77	47.8		
III	19	12.0	8	5.0		
Neoadjuvant therapy					0.8	0.99
Regimen A—oxaliplatin/capecitabine	93	58.9	-	-		
Regimen B—cisplatin/5-fluoruracil	55	34.8	-	-		
Regimen C—concomitant radiation	10	6.3	-	-		
Mandard response					-	-
1	13	8.2	-	-		
2	15	9.5	-	-		
3	29	18.4	-	-		
4	51	32.2	-	-		
5	50	31.6	-	-		
UICC stage preoperative					< 0.001	< 0.001
0	8	5.1	-	-		
1	13	8.2	24	14.9		
2	26	16.5	45	28.0		
3	90	57.0	78	48.4		
4	21	13.3	14	8.7		
ASA preoperative					0.86	0.44
1	38	24.1	22	13.7		
2	95	60.1	120	74.5		
3	22	13.9	17	10.6		
4	3	1.9	2	1.2		
Surgical approach					-	-
Abdominal	40	25.3	77	47.8		
Abdominothoracal	118	74.7	84	52.2		
(y) pT					< 0.001	< 0.001
0	14	8.9	-	-		
1	18	11.4	51	31.7		
2	29	18.4	55	34.2		
3	89	56.3	49	30.4		
4	8	5.1	6	3.7		
(y) pN					0.017	0.003
0	61	38.6	77	47.8		
1	57	36.1	55	34.2		
2	18	11.4	19	11.8		
3	22	13.9	10	6.2		
(y) G					< 0.001	< 0.001
0	12	7.6	-	-		
1	1	0.6	5	3.1		
2	58	36.7	75	46.6		
3	87	55.1	81	50.3		
Lymph node ratio (LNR, mean ± SD)	0.16 (± 0.22)		0.18 (± 0.25)		< 0.001	< 0.001
Adjuvant therapy					< 0.001	< 0.001
Yes	44	27.8	81	50.3		
No	114	72.2	80	49.7		
Relapse					-	-
Yes	96	60.8	113	70.2		
No	62	39.2	48	29.8		
Disease-free survival (DFS, median (IQR))	531 (217–2118) days		820 (209–2764) days		-	-
Death					-	-
Yes	106	67.1	125	77.6		
No	52	32.9	36	22.4		
Overall survival (OS, median (IQR))	972 (387–2323) days		1154 (358–2876) days		-	-

*BChE* butyrylcholinesterase, *SD* standard deviation, *HR* hazard ratio, *CI* confidence interval, *BMI* body mass index (kg/m^2^), *AEG* adenocarcinoma of the esophagogastric junction, *yT* pathologic tumor size, *yN* pathologic lymph node involvement, *G* tumor differentiation, *LNR* lymph node ratio, UICC stage, *ASA* American Society of Anesthesiologists physical status classification system

**Table 2:** Multivariable analyses estimating the influence of BChE and clinicopathologic parameters on disease-free survival (DFS) and overall survival (OS) for all patients, *n* = 319. Contrasts with discrete parameters were estimated with regard to grade/stage 1

Factors	*p* value (multivariate)	HR	95% CI
Disease-free survival (DFS)			
cT preoperative	0.31	-	-
cT2	0.05	0.46	0.21–1.00
cT3	0.19	0.54	0.21–1.37
UICC preoperative	0.62	-	-
UICC 2	0.13	1.78	0.84–3.77
UICC 3	0.16	1.99	0.76–5.19
UICC 4	0.12	2.30	0.81–6.54
Preoperative BChE			
Neoadjuvant chemotherapy	0.049	0.92	0.84–1.00
Primary surgery	0.35	0.96	0.87–1.05
(y)pT	0.11	-	-
(y)pT2	0.00	2.48	1.33–4.62
(y)pT3	0.00	3.37	1.51–7.52
(y)pT4	0.03	2.97	1.10–8.03
Grading (G)	0.57	-	-
G2	0.94	1.04	0.37–2.92
G3	0.76	1.18	0.42–3.33
LNR	< 0.001	4.99	2.52–9.90
Adjuvant chemotherapy	0.414	1.17	2.52–9.90
Overall survival (OS)			
cT preoperative	0.14	-	-
cT2	0.04	0.47	0.23–0.97
cT3	0.21	0.57	0.24–1.38
UICC preoperative	0.18	-	-
UICC 2	0.06	2.00	0.97–4.15
UICC 3	0.15	1.95	0.78–4.83
UICC 4	0.06	2.52	0.94–6.79
Preoperative BChE			
Neoadjuvant chemotherapy	0.049	0.92	0.85–1.00
Primary surgery	0.45	0.97	0.89–1.06
(y)pT	0.02	-	-
(y)pT2	< 0.001	2.71	1.51–4.85
(y)pT3	0.001	3.62	1.67–7.87
(y)pT4	0.028	2.98	1.13–7.89
Grading (G)	0.90	-	-
G2	0.85	0.91	0.36–2.32
G3	0.94	1.04	0.40–2.67
LNR	< 0.001	5.42	2.81–10.44
Adjuvant therapy	0.73	1.07	0.74–1.54

*BChE* butyrylcholinesterase, *HR* hazard ratio, *CI* confidence interval, *BMI* body mass index (kg/m^2^), *AEG* adenocarcinoma of the esophagogastric junction, *cT* clinical tumor size, *G* tumor differentiation, *LNR* lymph node ratio

**Table 3:** Backward regression analysis on the influence of BChE and clinicopathologic parameters on DFS and OS for all patients, *n* = 319

Factors	*p* value (multivariate)	HR	95% CI
Disease-free survival (DFS)			
(y)pT2	0.004	2.08	1.27–3.42
(y)pT3	< 0.001	3.27	2.00–5.35
(y)pT4	0.006	2.96	1.37–6.36
Preoperative BChE			
Neoadjuvant chemotherapy	0.04	0.92	0.84–1.00
Primary surgery	0.44	0.96	0.88–1.06
Lymph node ratio (LNR)	< 0.001	6.08	3.41–10.83
Overall survival (OS)			
(y)pT2	< 0.001	2.21	1.39–3.50
(y)pT3	< 0.001	3.37	2.11–5.39
(y)pT4	0.004	3.01	1.42–6.38
Preoperative BChE			
Neoadjuvant chemotherapy	0.05	0.92	0.85–1.00
Primary surgery	0.56	0.97	0.89–1.06
Lymph node ratio (LNR)	< 0.001	6.13	3.48–10.78

*BChE* butyrylcholinesterase, *HR* hazard ratio, *CI* confidence interval, *BMI* body mass index (kg/m^2^), *AEG* adenocarcinoma of the esophagogastric junction, *cT* clinical tumor size, *G* tumor differentiation, *LNR* lymph node ratio

## Discussion

Since BChE has been associated with malnutrition, chronic inflammation, and impaired liver function, as well as advanced tumor stage, it is a promising prognostic tool to consider during decision-making for treatment regimes in oncologic patients. Therefore, the current analysis aimed to evaluate the prognostic role of pretherapeutic serum BChE levels in patients with resectable adenocarcinoma of the esophagus and the gastroesophageal junction. We were able to identify a significant correlation of decreased pretherapeutic serum BChE levels in patients receiving neoadjuvant chemotherapy with shortened DFS and shortened OS, independent of several established prognostic factors. This represents the first retrospective analysis in a single center cohort study on the influence of preoperatively diminished BChE in patients with AEG over an observational period of almost 20 years. Since the established standard regimen for locally advanced AEG is the combination of neoadjuvant (radio-)chemotherapy and surgical resection [29, 30], a sub-analysis, investigating the role of BChE as prognostic factor in patients receiving neoadjuvant therapy with AEG, was performed. In our results, BChE was identified as an independent and prognostic marker not only in univariated analysis for all patients, but also in multivariate analysis for DFS and OS in patients with AEG after neoadjuvant therapy. Regarding current guidelines, patients with advanced tumor stage received neoadjuvant therapy. Amendments in UICC classification and therapeutic regimen were considered and adjusted to current guidelines within the observational period. Although a correlation of advanced tumor stage and diminished BChE was observed, UICC stage did not play a significant role in patient’s prognosis in our results. Furthermore, complete response to preoperative (radio-)chemotherapy of only 4.3% was clearly lower as reported in previously published studies [27, 31]. The observations we made in this study are similar to recently published data, investigating serum BChE levels in cervical cancer [21]. In that study, an association of decreased serum BChE levels with impaired DFS and OS and an indirect correlation to advanced tumor stage were observed in patients with cervical cancer. Not only an association of advanced tumor stage or enlarged tumor mass and chronic inflammation has been described in previous studies [32], but also an association of ongoing systemic inflammation and decreased serum BChE [22, 33]. Therefore, it indicates that decreased serum BChE levels may illustrate an ongoing chronic inflammatory reaction as a result of underlying and advanced malignancy. An analysis on the correlation of serum BChE and inflammatory markers in patients with malignancy might be of interest for further prospective investigations. Malnutrition, as a result of impaired nutritional intake, represents a negative prognostic factor in a variety of malignancies, esophageal cancer included. In a previously published investigation by our study group, not only malnutrition but also sarcopenia have been associated with shorter survival in patients with esophageal cancer [34]. Our results demonstrated a tendency of decreased BChE and diminished preoperative mean BMI, especially in patients receiving neoadjuvant therapy. In univariate analysis, diminished BMI indicated a trend to shortened DFS (*p* < 0.05) and OS (*p* < 0.17) in all patients (Table 1). Preoperative ASA classification as a potential indicator for patients’ health condition did not show a significant association regarding DFS and OS (Table 1). Due to the retrospective study setting, it unfortunately was not possible to identify whether patients underwent preoperative dietary measurements such as pre-habilitation and carbo-loading, which nowadays are part of our clinical routine, naturally. Since patients receiving neoadjuvant therapy are more often diagnosed in advanced tumor stages, the influence of systemic cytostatic therapy on potentially impaired liver function, reduced physical strength, or tumor cachexia might be another possible explanation for worse outcome in those with diminished BChE. In our results, a decrease of serum BChE in patients receiving neoadjuvant treatment (group A) within the observational period was noticed. Nevertheless, serum BChE in patients undergoing primary resection (group B) was significantly lower prior to surgery. Both observations might be correlated to disease-specific symptoms, continuous malnutrition, and reduced oral food intake, as well as diminished physical state, subsequently leading to impaired OS and increased mortality [35, 36]. Since an association of decreased serum BChE levels and malnutrition independent of cancer disease was described previously, further evaluation of chronic inflammation, malnutrition, and the association of serum BChE levels in tumor patients would be of interest [37, 38]. As in other retrospective studies, benefits but also limitations must be pointed out. The consistent patient’s management by a well-trained, specialized team of general surgeons, involving making a diagnosis, discussing treatment options in an interdisciplinary team according to guidelines, and initiating further therapy to postoperative aftercare, has to be mentioned as the main benefit of this study. Our result regarding general patient survival was 35.4 months and similar to published results regarding 3-year OS [39]. Within the observational period of more than 2 decades, all patients who were resected due to an AEG were included in this analysis. If values regarding BChE or patient’s follow-up were missing in this retrospective analysis, patients had to be excluded. Although the correlation of diminished BChE with patient’s outcome was very strong, we could not define an ideal statistical and clinically relevant cut-off parameter yet. In general, the process of blood sampling and analysis of BChE is simple and did not change within the observational period; therefore, laboratory results were robust and can doubtlessly be used for analysis. However, since the current laboratory assessment for BChE might not adequately reflect the importance of decreased BChE levels in clinical practice, we are currently investigating additional methods to better specify the levels of BChE and define an adequate cut-off parameter. Although adaptions had to be performed according to current guidelines and classifications for the analysis, changes in therapeutic pathways such as surgical technique and medical treatment still might be influencing our results and data interpretation on OS and DFS—even though neoadjuvant therapy and surgical approach did not show a significant correlation in our results. Since our analyses show similar results as other trials with a similar analysis in differing malignancies, the mentioned limitations might have limited effect on our results. As BChE is part of the routine blood analysis, it represents a cost-effective biomarker. Nevertheless, the underlying pathway for the development of diminished BChE in malignant disease and therefore the prognostic role of BChE in AEG still is not fully understood. Since previous studies described an association with various epithelial malignancies, observing the role of BChE in patients with esophageal squamous cell cancer and comparing the results to those with AEG patients might be of interest in further analysis [40]. Based on our results, serum BChE might find recognition in the clinician’s daily routine, identifying patients at risk for reduced therapeutic or postoperative outcome and subsequently elevated risk for early recurrence and diminished overall survival. It might be a safe, easily accessible, and cost-effective screening tool for patient’s outcome, especially for those in need of neoadjuvant therapy. After validation of our results in further prospective studies, pretherapeutic BChE could be used for patient counseling and guidance in treatment decision-making. To conclude, this analysis indicates the efficiency of diminished pretherapeutic serum BChE as a potential novel independent prognostic biomarker in patients with AEG after receiving neoadjuvant therapy.
